# Virtual Screening of Novel Benzothiozinone Derivatives to Predict Potential Inhibitors of Mycobacterium Tuberculosis Kinases 2D-QSAR, Molecular Docking, MM-PBSA Dynamics Simulations, and ADMET Properties

**DOI:** 10.3390/ijms26115129

**Published:** 2025-05-27

**Authors:** Abdelmadjid Guendouzi, Lotfi Belkhiri, Zakaria Slimani, Abdelkrim Guendouzi, Gautier Moroy

**Affiliations:** 1 Higher Normal School of Constantine ENS Assia Djebar Constantine, Ali Mendjeli, Constantine 25000, Algeria; guendouzi.abdelmadjid@ensc.dz; 2 Pharmaceutical Sciences Research Center CRSP, ZAM Ali Mendjeli, Constantine 25000, Algeria; 3 Laboratory of Mathematical and Subatomic Physics LPMS, University of Constantine 1 Frères Mentouri, Constantine 25017, Algeria; 4 Faculty of Life Sciences and Nature, Hassiba Benbouali University of Chlef-UHBC, Ouled Farès 02000, Algeria; sanslimz@gmail.com; 5 Theoretical Physics Laboratory, Abou Bakr Belkaid University of Tlemcen, Tlemcen 13000, Algeria; 6 Laboratory of Chemistry, Synthesis, Properties, and Applications (LCSPA), University of Saïda, Saïda 20000, Algeria; abdelkrim.guendouzi@univ-saida.dz; 7 Unité de Biologie Fonctionnelle et Adaptative, BFA, CNRS UMR 8251, INSERM ERLUniversité Paris Cité, U1133, F-75013 Paris, France

**Keywords:** anti-tuberculosis, mycobacterium, benzothiozinone, in silico, virtual screening, 2D-QSAR, molecular docking, molecular dynamics simulations, ADMET

## Abstract

Mycobacterium tuberculosis, the infectious agent behind tuberculosis (TB), underscores the significance of targeting enzymes such as arabinosyltransferases in drug development efforts. Benzothiozinone derivatives, which have been assessed for their effectiveness against TB, present a promising avenue for treatment. Utilizing a high virtual screening quantitative structure–activity relationship (QSAR-VS), a set of forty Benzothiozinone (C1–C40) compounds were investigated to build a robust model with satisfactory performance metrics (*R*^2^ = 0.82, *R*^2^*_adj_* = 0.78, *N_test_* = 10, *R*^2^*_test_* = 0.70). This model enabled the creation of databases containing new derivatives for screening drug-like properties and predicting MIC activity in TB treatment. The best-scoring compounds were screened by molecular docking with Mycobacterium tuberculosis kinases A and B (PDB code: 6B2P) and validated by molecular dynamics simulations to elucidate the most stable drug–protein interactions. Additionally, the MM-PBSA analysis shows that the strongest binding occurs in complexes X3, X4, and X6 with Δ*G_bind_* values of −8.2, −15.3, and −12.0 kcal/mol, respectively. Our in silico study aims to prospect these new anti-tubercular drugs and their potential development through perspective in vitro and in vivo assays.

## 1. Introduction

As reported, tuberculosis remains one of the primary infectious diseases globally, leading to more than 10 million active cases annually and causing around 1.5 million deaths each year [1,2]. Mycobacterium tuberculosis (Mtb) is the main causative agent of tuberculosis, leading to the emergence of multidrug-resistant (MDR) and extensively drug-resistant (XDR) strains of Mtb, as well as treatment failures in tuberculosis patients [3]. The mycobacterial cell envelope, with its intricate and unique structure, has been identified as a promising target for developing novel tuberculosis drugs [3,4,5,6,7,8]. This envelope consists of layers such as peptidoglycan, mycolic acids, and arabinogalactan polysaccharides, which play a critical role in protecting and supporting Mtb growth [3]. Researchers are exploring enzymes involved in cell wall biosynthesis and other potential drug targets within this pathway to develop more effective strategies for new anti-tuberculosis drug development [3,4].

**Figure 1:**
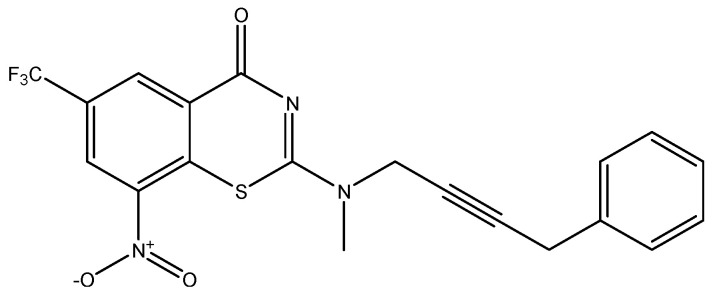
Molecular structure of the reference C11 derivative.

Developing new and more effective agents to combat the global tuberculosis epidemic by targeting the essential proteins of resistant Mtb is a promising strategy [9]. Notably, a recent review on anti-tuberculosis drug development via targeting the cell envelope of Mtb was reported, summarizing the development of drug enzymes involved in the biosynthesis of the cell wall in Mtb, and other potential drug targets in this pathway, providing more effective strategies for the development of new drugs [10]. Moreover, Xiaomei et al. [11] reported a structure–activity relationship (SAR) analysis on a series of novel forty Benzothiozinone (btz) derivatives (C1–C40) as potent inhibitors for Mtb kinases. The SAR analysis aimed to identify the pharmacophore groups inhibiting tubercular activity and led to the discovery of novel agents with high minimal inhibitory concentration (MIC) with improved metabolic stability. As reported, the compound C11, shown in Figure 1, demonstrated the best antimycobacterial activity in an acute tuberculosis infection mouse model [11].

Despite much effort to identify efficient drug sensitivity towards tuberculosis, the latter remains a significant global health challenge, and innovative approaches are essential to reduce MDR strains and improve anti-tuberculosis treatment outcomes. In recent years, in silico techniques involving computational and analytical methods have become increasingly prominent for designing and discovering novel compounds with noteworthy biological properties [12].

Moreover, the drug discovery process for Mtb has been enhanced with a structure-based computer-aided drug design (CADD) approach [3]. This CADD approach, including quantitative structure–activity relationships (QSAR), molecular docking, molecular dynamics simulations, and ADMET properties, combined with advanced software are currently used to develop, predict, and optimize active compounds (hits) as potential inhibitors against tuberculosis resistance and can be utilized in future chemotherapeutic development [4].

In the present study, in silico approaches were used on forty Benzothiozinone derivatives (C1–C40), reported recently as effective agents against Mycobacterium tuberculosis kinases [11], to predict novel potential inhibitors. The two-dimensional 2D-QSA model combined with the multiple linear regression (MLR) technique was used to design and evaluate new candidate compounds with improved tuberculosis inhibitory activity compared with the actual in vitro tested agents [11]. A new data library based on the modified synthesized series was also used to predict the minimal inhibitory concentration (pMIC) values. A molecular docking technique is employed to identify selected active amino acid residue sites within the tubercular target, with the stability of various ligand poses analyzed using molecular dynamics (MD) simulations. Finally, the drug-likeness of selected hits is assessed using the ADMET profile.

## 2. Results and Discussions

Several steps were involved to predict compounds with potent anti-tuberculosis activity to narrow down the potential inhibitor compounds. First, ADMET prediction screening evaluates the compounds’ adherence to Lipinski’s rules and pharmacokinetic properties. Subsequently, the 2D-QSAR model is used to predict the anti-tuberculosis activities within the value range of pMIC ≥ 7.5. The final screening of Mtb inhibitors involves molecular docking followed by dynamics simulations.

The SwissADME and pkCSM platforms are used to evaluate ADME properties, including Absorption, Distribution, Metabolism, and Excretion. Subsequently, the filtering is based on Lipinski’s Rule of Five, i.e., molecular weight (MW ≤ 500 Da), rotatable bonds (RTB ≤ 12), hydrogen bond donors (HBD ≤ 5), hydrogen bond acceptors (HBA ≤ 10), and Lipophilicity factor (LogP < 5) should be accounted to ensure a robust database. Additionally, compounds with predicted pMIC values lower than 7.5 were excluded. Compounds not meeting these criteria were excluded from the 2D-QSAR modeling and docking studies [13,14,15,16,17].

### 2.1. Two-Dimensional QSAR Analysis

A genetic algorithm for descriptor selection is employed to develop a robust 2D-QSAR model for Mtb inhibition activity. Several 2D-QSAR models of pMIC activity were generated and validated to assess their capability to predict and enhance the biological activities of novel compounds. The selected pMIC model is formulated by the following equation:

pMIC = −0.54 × *nN* − 55.69 × *PW2* + 0.15 × *RDF100m* + 24.61 × *G2m* + 7.88 × *R8u* + 32.39 (1)

where descriptors are defined as follows:

*nN*: number of Nitrogen atoms;

*PW2*: path/walk 2-Randic shape index;

*RDF100m*: Radial Distribution Function-100/weighted by mass;

*G2m*: 2nd component symmetry directional WHIM index/weighted by mass;

*R8u*: R autocorrelation of lag 8/unweighted.

During the training process, which utilizes 30 data points, the model yields the following statistical metrics:

Number of testing data points *N_test_* = 12, *R*^2^ = 0.81, *R*^2^*_adj_* = 0.78, *MSE* = 0.15, *Q*^2^*_CV_* = 0.69, *R*^2^*_test_* = 0.8, and *Y_rand_* = 0.22.

These statistical results indicate that the selected model surpasses threshold values for all parameters, in agreement with previous findings [18,19].

The prediction reliability of the developed MLR 2D-QSAR model is validated by metrics aligning with benchmark scores proposed by Golbraikh and Tropsha (2010) [20,21]. The determination coefficient assessment *R*^2^ value, exceeding 0.5, underscores the robustness of the 2D-QSAR model and its resistance to chance correlation during the modeling process [4,19,22,23].

Indeed, the determination coefficient (*R*^2^ = 0.81) suggests that the 2D-QSAR model accounts for 81% of the total variation in biological activities, demonstrating its satisfactory reliability for the training set compounds. Furthermore, the higher *R*^2^ value emphasizes that the model’s performance is not merely a result of chance correlation. Oppositely, low *R*^2^ and *Q*^2^*_CV_* values obtained from shuffled biological activities suggest the model’s inadequacy in creating a reliable predictive model under such conditions.

The QSAR-MLR multilinear regression analysis was carried out to showcase the correlations between selected descriptors and observed Mtb biological activity (pMIC), and the results are reported in Table 1.

**Figure 2:**
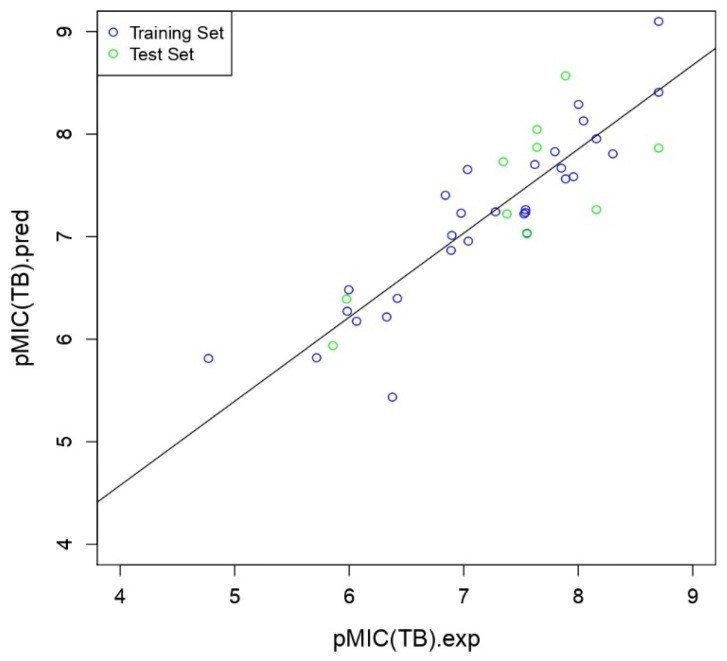
Correlation of observed and predicted pMIC values in the 2D-QSAR models.

A comparison between predicted pMIC (TB) values and experimental pMIC (TB) values is illustrated in Figure 2. The data points are divided into the training set (blue dots) and the test set (green circles).

A line of best fit runs through the data points, indicating the relationship between the predicted and experimental values. Points closer to this line represent more accurate predictions. This visual representation helps to assess the model’s performance in predicting biological activity, which is crucial for drug design and discovery.

**Figure 3:**
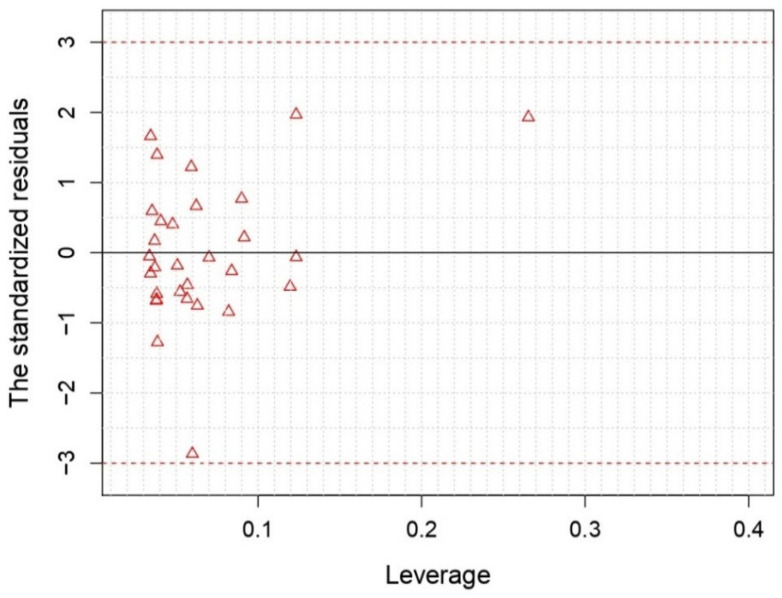
Visualization of the Williams plot depicting standardized residuals versus leverage for the MLR model, with h * = 0.250 and residual limits of ±3.

Figure 3 shows the Williams plot, which plays a crucial role in establishing the applicability domain (AD) of the QSAR model. By assessing the standardized residuals against leverage values, the plot helps identify outlier compounds and ensure the model’s reliability.

With five descriptors, the developed 2D-QSAR model excludes outlier compounds by setting a normalized residue limit of 3. All compounds in the training and test sets fell within the AD, as indicated by leverage values below the critical limit and standardized residuals within the range of ±3, which further validates the robustness of the model.

### 2.2. Library Creation

To optimize the leads for targeting specific biological activities, our 2D-QSAR approach involves developing a compound library by replacing ‘R’ atoms with distinct chemical moieties (see Figure 1). Utilizing a predefined R package library, it results in approximately 8^28^ compounds, and each structure underwent energy minimization to ensure optimal alignment. Furthermore, to ensure a robust database, primarily filtering was applied for virtual screening, including ADMET criteria, which allow excluding molecules with pMIC values lower than 7.5.

### 2.3. Virtual Screening and Molecular Docking

After the pMIC threshold (>7.5) and ADMET filters, a high virtual screening (HVS) using the molecular docking technique was performed with the Molegro program to focus on the 10% most promising newly designed (X1–X20) molecules and gain more precise insights into their interaction with the biological target [11].

**Figure 4:**
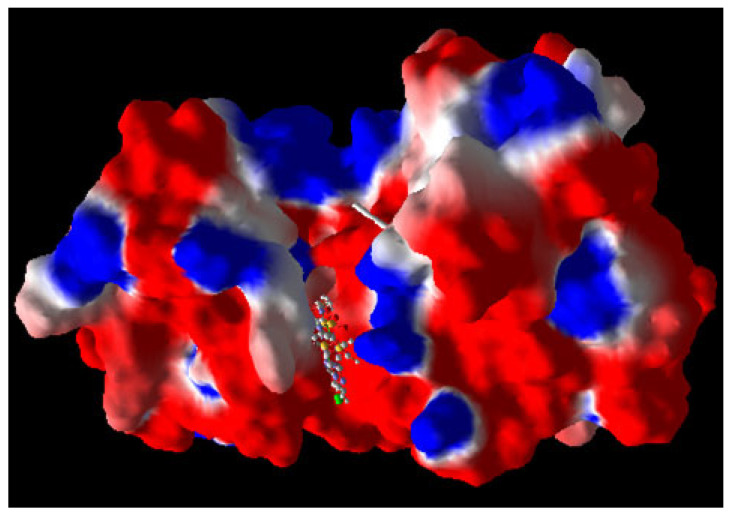
Conformational relationship between the pose and its reference ligand in the inhibitor 6B2P pocket (RMSD = 1.5 Å).

The re-docking experiment was conducted on the protein structure, and the result is depicted in Figure 4.

Notably, the best pose obtained resulted in an RMSD value of 1.5 Å for kinases A and B in the Mtb 6B2P target. These results indicate that the re-docking test successfully reproduces the active site of the crystal reference ligand pose within the 6B2P protein.

For the assessment of their affinity, all designed compounds were docked against the Mtb kinase, and the best conformations in the binding energy of Mtb and hydrogen bond energy (kcal/mol) results, are reported for the twenty selected (X1–X20) ligands and the native compound in Table 2 (the other compounds are reported in Table S1).

Notably, the complexes formed between the designed compounds and the active residues of the Mtb kinase exhibit negative and low-binding energies, implying that the inhibition of tuberculosis is a thermodynamically favorable process. Indeed, MolDock score values (kcal/mol) of the top twenty (X1–X20) selected molecules range between −153.5 and −140.3 kcal/mol.

The three best-scoring ligands (X1, X2, and X3) show the highest binding stability with the MolDock score order (kcal/mol) of X1 (−153.5) > X2 (−152.8) > X3 (−152.1). These results (Table 2) strongly support the ability of the selected designed compounds (X1–X5) to bind the active site of the Mtb target and act as effective anti-tuberculosis inhibitors.

**Figure 5:**
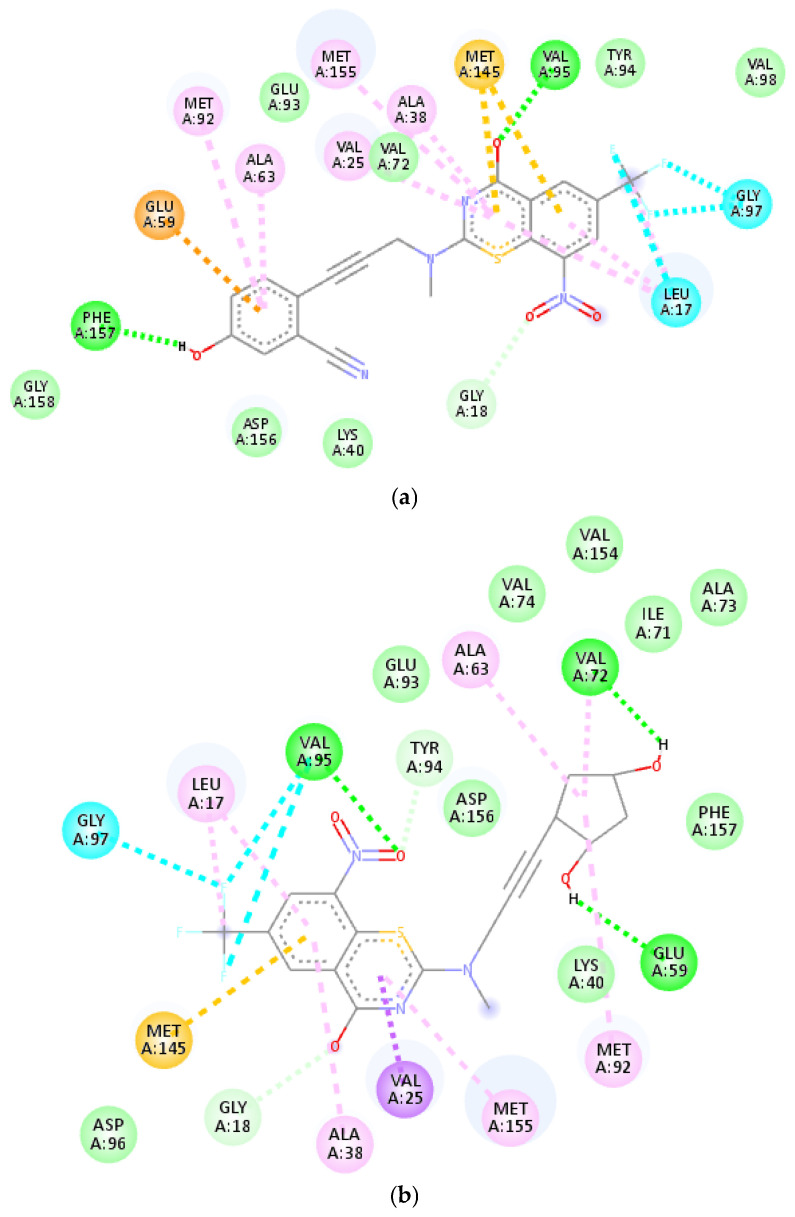

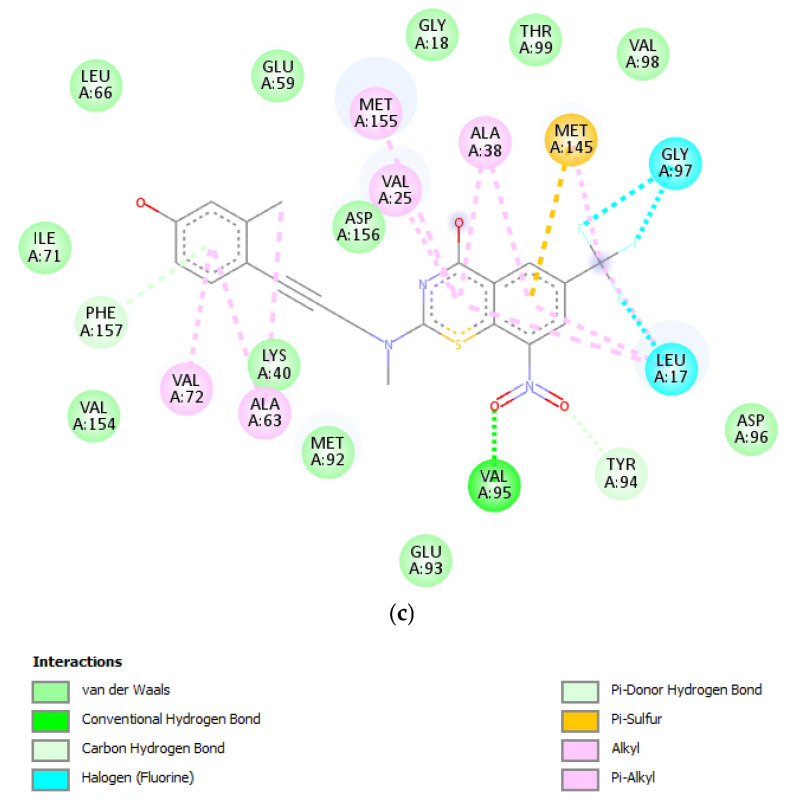
Two-dimensional interactions between active site residues of the tuberculosis kinase and compounds (**a**) X1, (**b**) X2, and (**c**) X3.

Their 2D interaction profiles for the three top-scoring ligands (X1, X2, and X3) with the active site residues are illustrated in Figure 5, highlighting key binding interactions within the active Mtb site. According to the 2D structural model generated via Biovia Studio, the active site of the tuberculosis kinase 6B2P target was identified.

As shown in Figure 5, the first best-docked X1 ligand, showing the higher energy score (−153.5 kcal/mol), binds to the target protein and establishes two conventional H-bonds (green dotted lines) with PHE A: 157 and VAL A: 95 amino acid residues. Additional amino acid residues involved in molecular interactions with X1 include GLU A: 59, MET A: 145, GLY A: 97, LEU A: 17, ILE A: 78, VAL A: 167, and ILE A: 94.

Moreover, the second best-scoring X2 ligand, totalizing binding energy of −152.8 kcal/mol, establishes three hydrogen bonds with GLY A: 59 and VAL A: 95 amino acid residues within the kinase 6B2P protein. In addition, multiple interactions including π–alkyl, π–sigma, and π–sulfur bonds occur with LEU A: 17, MET A: 92, MET A: 155, ALA A: 38–63, and ILE A: 78 residues, contributing to the binding energy score (−152.8 kcal/mol), which closely aligns with that of the X1 congener.

The third best-scoring X3 ligand binds to the target protein, forming one conventional H-bond with VAL A: 95 residues. Furthermore, X3 interacts with other amino acid residues such as GLU A: 50, ILE A: 78, VAL A: 167, and ILE A: 94, featuring a binding affinity of −152.1 kcal/mol.

The molecular docking results (Table 2) of the newly designed best three (X1–X3) compounds reveal that binding affinity scores are relatively close, which suggests that these compounds might have a similar ability to inhibit the 6B2P target kinase.

### 2.4. Molecular Dynamics (MD) Simulations

Molecular dynamics (MD) simulations are widely used in various biological applications to simulate structural and physiological perturbations, providing close real-time mobility of complexes [4,19,24,25,26]. The trajectories obtained from these simulations offer valuable and detailed insights into the stability of ligand–receptor complexes and their molecular interactions.

**Figure 6:**
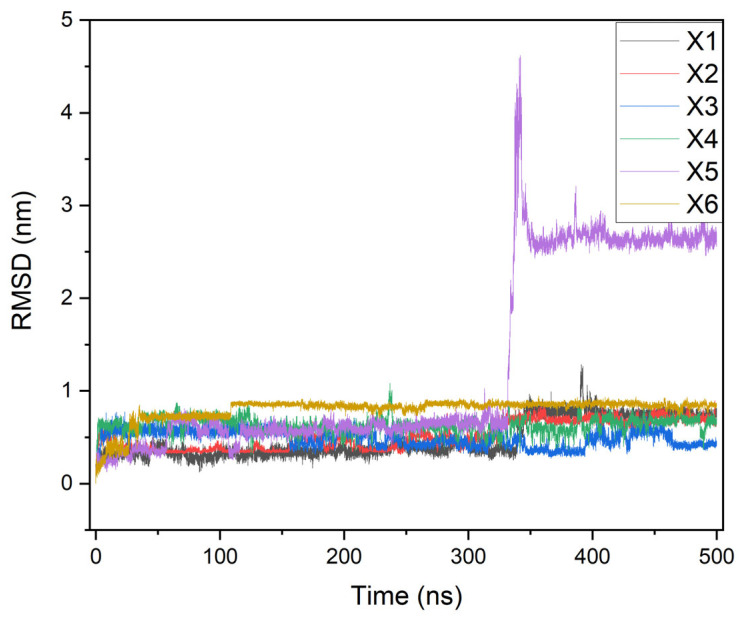
RMSD maps of the receptor–ligands at 500 ns of simulation time.

**Figure 7:**
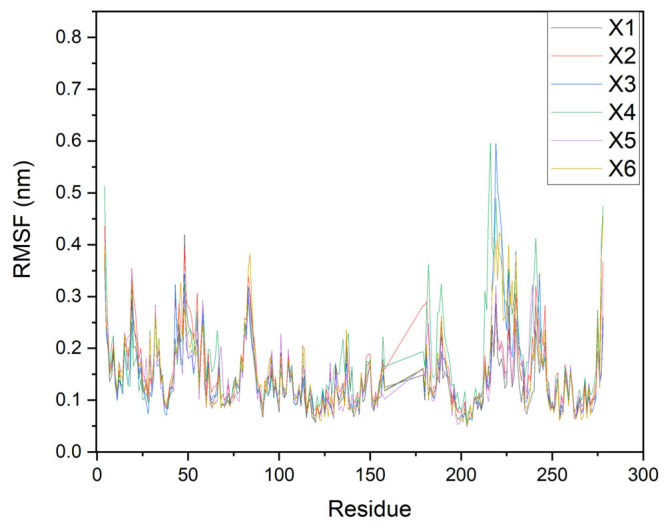
RMSF of protein and the protein–ligand complexes.

**Figure 8:**
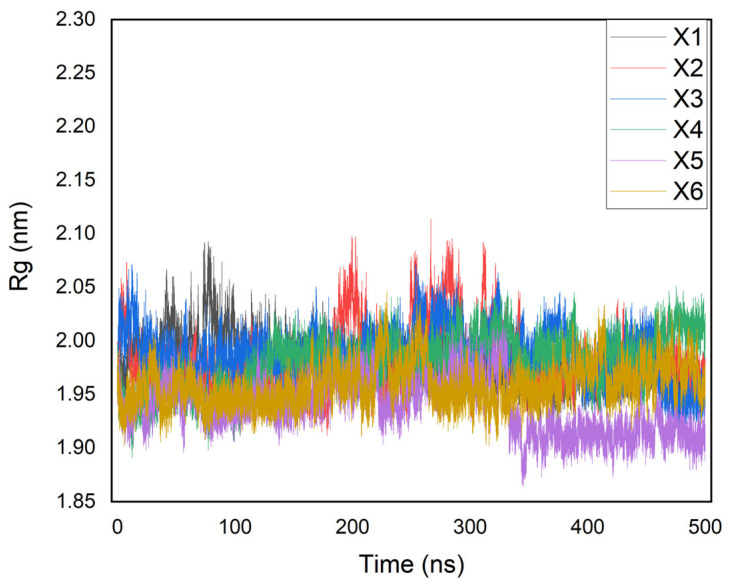
Rg of proteins and ligands.

The dynamics results (500 ns) of the high-scoring ligands (X1–X6) with tuberculosis kinase targets are performed using the RMSD, RMSF, and Rg parameters, as depicted in Figure 6, Figure 7 and Figure 8, respectively.

Figure 6 shows the variations in the RMSD values obtained for the complexes (X1–X6) associated with the tuberculosis kinase 6B2P, except for the X5 system, which oscillates between 1.8 and 2.8 Å and reaches stability after the first 20 ns of simulation. It is noteworthy that the RMSD for the free protein 6B2P is observed to range over 10 Å [19,22,27,28].

Additionally, the RMSF analysis shown in Figure 7 represents the average deviation of the residues over the 500 ns simulation from a reference position. RMSF values also indicate the thermodynamic stability and mobility rate of all residues. Figure 7 shows that RMSF values for protein–ligand complexes are less than 0.5 nm, suggesting that the ligands do not undergo significant conformational changes over time, despite the presence of different amino acids in the systems. Higher values indicate variable regions in proteins, while lower values indicate fixed regions [19,27].

Additionally, according to the MD simulations, the stability of the selected protein–ligand complexes over 500 ns is supported by the Rg data, as shown in Figure 8.

Indeed, the Rg parameter remains consistent with an average value of 1.9–2.1 nm, indicating both the dimensional stability and the compactness of the structures. Notably, a stably folded protein maintains relatively less variation in Rg values, signifying its dynamic stability. Furthermore, the Rg values suggest that the high-scoring ligands (X1–X6) remained stably bound to the 6B2P protein, without inducing any significant alterations in their structures [19,25,28].

The Δ*G* binding free energy (Δ*G_bind_*) of the best-scoring compounds with the protein 6B2P was calculated from the last 100 ns MD simulation trajectories through the MM-PBSA approach. The calculated average Δ*G_bind_* values and standard deviations are given in Table 3.

Table 3 shows that the strongest binding occurs in complexes X3, X4, and X6 with Δ*G_bind_* values of −8.2, −15.3, and −12.0 kcal/mol, respectively. These complexes also have the strongest van der Waals (vdW) and electrostatic interactions, contributing significantly to their stability. Oppositely, X5 shows the weakest and most highly unfavorable binding with a Δ*G_bind_* of +22.8 kcal/mol, likely due to unfavorable entropic effects or solvation penalties, despite moderate vdW (−25.9 kcal/mol) and electrostatic (−10.5 kcal/mol) attractive terms. Moreover, the X2 and X1 compounds exhibit weak to slightly favorable binding. Overall, van der Waals interactions are the dominant factors in stabilizing most complexes, as these contributions are consistently more negative than the electrostatic energies.

## 3. Materials and Methods

### 3.1. Building the Compound Dataset

**Figure 9:**
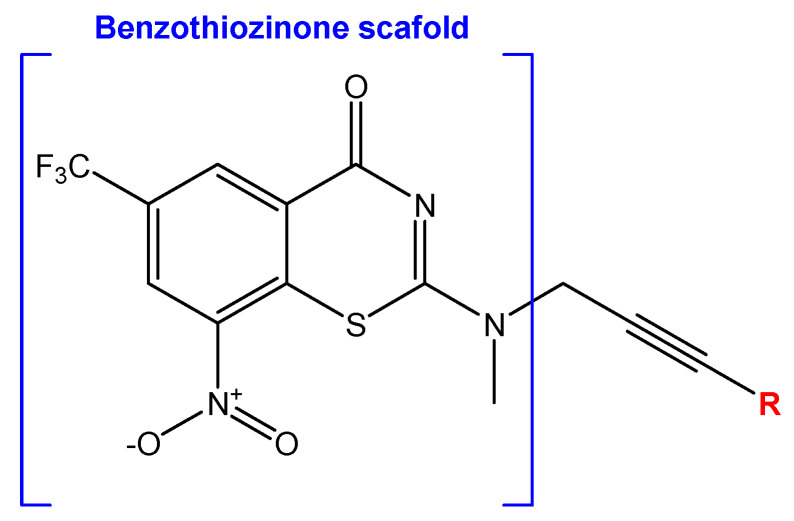
Molecular structures of BTZ derivatives.

To computationally evaluate the anti-Mycobacterium tuberculosis activity of new agents against the target kinases, the 2D-QSAR model was first built for the selected forty Benzothiozinone derivatives (C1–C40) compounds with their substituted structures (R) as shown in Figure 9, and their molecular structures are gathered in Table 4. As shown in Figure 1, the Benzothiozinone scaffold is kept fixed, while the alkynyl linker (R) is varied.

Employing the 2D-QSAR model with Python 3.9.10, a database comprising Benzothiozinone derivatives was curated with compounds C1 and C11 demonstrating high affinity for the desired receptor and low to moderate activity for off-target receptors. The forty molecules were divided into a test set (*) of 10 compounds and a training set comprising the remaining molecules.

All compounds were imported into DRAGON v5.4 [29], and among 1665 descriptors, 20 types were selected, mainly providing structural and contact information.

### 3.2. Two-Dimensional QSAR Virtual Screening and Model Development

To investigate the association between a dependent variable, specifically anti-tuberculosis activity, and a set of selected descriptors (independent variables), we conducted a virtual screening 2D-QSAR-VS analysis using Dragon and Molinspiration descriptors within the environment of R Studio version 2023.12.1 Build 402 (Posit Software, PBC) [30]. MLR technique was applied to the forty Benzothiozinone derivatives to assess the statistical robustness of the 2D-QSAR model. Furthermore, metric parameters such as the determination coefficient (*R*^2^), adjusted coefficient (*R*^2^*_adj_*), and mean squared error (MSE), in addition to the Y-randomization (*R*^2^*_rand_*) and leave-one-out (LOO) cross-validation of the 2D-QSAR-MLR model, are assessed.

The regression equation employed in our analysis is as follows [31]:

(2) $$\mathrm{pMIC}=B_{0}+B_{1}x_{1}+B_{2}x_{2}+\ldots +B_{n}x_{n}$$

The pMIC represents the dependent variable, *x_i_* denotes the independent variables (molecular descriptors), and *B_n_* represents the regression coefficients.

Moreover, the predictive performance of the 2D-QSAR-MLR model is evaluated using the *R*^2^*_test_* coefficient, which compares the observed and projected pMIC values for the test set. To minimize the risk of chance correlation, the Y-randomization test (*R*^2^*_rand_*) is conducted to ensure that the selected descriptors are not randomly correlated with the activities.

Additionally, the applicability domain (AD) is applied by using William’s plot and the R Studio software 2023.12.1 to assess leverage values and determine if a compound fell within the prediction boundaries of the model [18,20,32]. The AD approach was defined within a squared area, using a standard deviation (x = ±3) as a cutoff value for accepting predictions, thus enhancing confidence in the practical utility of the model’s prediction.

### 3.3. Virtual Screening

To build the chemical compound library, a Python script was developed and tailored to streamline the generation of a diverse range of compounds. This was carried out by systematically substituting the initial Benzothiozinone derivatives [33] and utilizing the RDKit library within the Python script for the efficient processing of chemical structures [34]. Initially, different substituents were introduced across forty different sites within the structures. Subsequently, energy minimization was carried out using RDKit’s MMFF force field [34], and the optimized resulting structures were stored for further analysis.

Based on Benzothiozinone derivatives, the tuberculosis library database was screened using Molegro Virtual Docker (MVD) version 6.0 [35]. The molecular docking of the ligands within the active sites of the protein kinases was carried out to identify optimal conformations for the ligand–protein complexes [36].

The re-docking test of the co-crystallized reference ligand is performed to validate the molecular docking analysis while keeping the protein structure unchanged with respect to its crystal-binding pocket. The poses obtained from the docking procedure were evaluated by comparing them to the crystal structure using the root mean square deviation (RMSD) parameter with the superpose option in Molegro. A docking technique is considered acceptable if the RMSD range is less than or equal to 2 Å [37]. The coordinates (x = 86.6, y = 76.5, z = 27.0) with a radius of 15 Å were defined as the docking site of the designed compounds in the protein 6B2P pocket. The virtual screening module was set to select the top 10% of the screened compounds with significant binding scores [38]. Specifically, the most active and least active molecules, along with designed drug candidates, were docked into the active site of dual inhibition of the essential protein Kinases A and B in the Mtb (PDB: 6B2P) [39], obtained from the RCSB website (https://www1.rcsb.org, accessed on 10 March 2024) [40].

Subsequently, small molecules with the highest docking scores were chosen for subsequent molecular dynamics simulations and Molecular Mechanics Poisson–Boltzmann Surface Area (MM-PBSA) analysis [41,42]. Discovery Studio Visualizer 2021 Client v21.1.0. was used to visualize different types of H-bond interactions within the ligand–protein complex [43].

### 3.4. Molecular Dynamics Simulations

To assess the stability of the best dock–scoring complexes, molecular dynamics (MD) simulations were performed using the GROMACS 2023.4 (released 24 January 2024) software [44]. The topology of the ligands was generated using the CHARMM force field implemented in the SwissParam tool [45,46]. The protein complex (PDB ID: 6B2P) was solvated in a cubic box using the SPC/E extended simple point charge water models [47], and counter ions were introduced to maintain the overall electrostatic neutrality of the system.

The simulation process involves several steps, including (1) reading the coordinates of the protein–ligand complex; (2) solvating the complex, determining the system’s shape and size, and neutralizing it by adding Na^+^ and Cl^−^ ions; (3) setting periodic boundary conditions (PBC) to simulate an extensive system, followed by a short minimization to remove erroneous contacts; (4) system equilibration in NVT and NPT ensembles to achieve the desired temperature and pressure [48]; and (5) a production phase involving MD runs with specific parameters.

Furthermore, energy minimization utilizes the steepest descent algorithm, with a maximum of 100.000 steps, until structures are stabilized to a maximum force of 1000 kJ.mol^−1^ nm^−1^. Subsequently, 5 ns of NVT and NPT equilibrations was performed at 300 K and 1 atm [48]. Simulations ran for 500 ns with a time step of 2 fs.

The dynamics parameters, i.e., the RMSD, the root mean square fluctuation (RMSF), and the radius of gyration (Rg) parameters are calculated over the 500 ns simulation period. MD simulation trajectories were visualized using PyMOL software (Schrödinger, LLC, version 2.5.7) [49]. The MM-PBSA approach is commonly used for estimating the binding energy of protein–ligand. In this context, the gmx_MM-PBSA tool developed by Valdes-Tresanco [41,42] was utilized to calculate binding free energies of the complexes using a single trajectory protocol from GROMACS MD trajectories. Entropy calculations were performed over the specified trajectory range (startframe = 30,734 to endframe = 49,734), corresponding to the time interval of 307.34–497.34 ns, using a frame extraction interval of 100 ps. The system temperature was set to 310.15 K, and a segment size (ie_segment) of 50 was applied. The binding free energy was computed with an electrolyte strength of 0.15 M and a grid fill ratio of 4.0 to solve the Poisson–Boltzmann equation efficiently.

## 4. Conclusions

In this work, an investigation of the anti-tuberculosis (pMIC) activity of forty Benzothiozinone derivatives (C1–C40) as potent inhibitors of Mycobacterium tuberculosis (Mtb) kinases was carried out by combining multiple in silico approaches, including 2D-QSAR, molecular docking, MM-PBSA dynamics simulations, and ADMET properties. The in silico study aimed to design and explore novel agents with better-predicted minimal inhibitory concentrations (pMICs) than the reference compound C11, which exhibits the best antimycobacterial inhibition activity in an acute tuberculosis infection mouse model. The developed 2D-QSAR MLR model was then used to design a new dataset of Benzothiozinone derivatives based on optimizing their ADME properties. Twenty (X1–X20) models were selected for their higher predicted inhibition activity (pMIC) in comparison with the best in vitro C11 derivative. The molecular docking analysis of the selected ligands for their binding interactions unveiled the best-scoring compounds (X1–X6). This suggests that they can effectively interact with the active residues of Mtb kinase as promising anti-tuberculosis inhibitors. Furthermore, their stability within the target Mtb protein was confirmed through RMSD, RMSF, Rg, and MM-PBSA dynamics simulation parameters. Based on these results, compounds X3, X4, and X6 emerge as promising drug candidates for tuberculosis treatment. Our in silico findings could pave the way to further research in developing and rationalizing anti-tuberculosis inhibitor drugs guided by their in vitro and in vivo assays.

## Figures and Tables

**Table 1:** Comparison of experimental and predicted activities pMIC, with (^★^) denoting test set samples.

ID	Exp	MLR	ID	Exp	MLR	ID	Exp	MLR	ID	Exp	MLR
1	8.15	7.95	11 ^★^	7.55	7.02	21	6.06	6.17	31	7.55	7.03
2	7.95	7.58	12	6.88	6.86	22	5.97	6.27	32	7.53	7.23
3 ^★^	7.88	8.56	13	7.52	7.21	23	4.77	5.81	33	7.61	7.70
4	7.88	7.56	14	7.03	6.95	24 ^★^	5.85	5.93	34 ^★^	7.34	7.73
5 ^★^	8.15	7.26	15	6.83	7.40	25	8.30	7.80	35 ^★^	8.69	7.86
6	7.03	7.65	16	6.32	6.21	26	8.69	9.10	36	8.04	8.12
7	7.53	7.26	17	5.99	6.47	27 ^★^	7.63	7.86	37	8.69	8.40
8	7.27	7.24	18	6.37	5.43	28	6.42	6.39	38	8.00	8.28
9	7.79	7.83	19	5.71	5.81	29	6.89	7.01	39 ^★^	7.63	8.04
10	7.85	7.66	20 ^★^	5.97	6.39	30	6.97	7.22	40 ^★^	7.37	7.22

**Table 2:** MolDock score and H-bond energy of the twenty best conformations (X1–X20) and the native compound in the binding pocket of Mtb.

Ligand	MolDock Score kcal/mol	H-Bond kcal/mol
X1	−153.5	−4.9
X2	−152.8	−7.1
X3	−152.1	−4.2
X4	−150.7	−5.5
X5	−150.7	−2.5
X6	−150.3	−7.2
X7	−149.9	−6.2
X8	−148.7	−5.6
X9	−148.2	−2.5
X10	−148.1	−3.4
X11	−146.5	−2.5
X12	−146.3	−3.8
X13	−146.2	−0.3
X14	−146.0	−3.8
X15	−146.0	−6.9
X16	−145.2	−6.5
X17	−145.1	−1.5
X18	−143.1	−2.5
X19	−142.0	−3.5
X20	−140.3	−2.4
Native	−131.9	−2.5

**Table 3:** Calculated binding free energies of the best compounds.

Compounds	Δ*G_bind_* kcal/mol	vdW Energy kcal/mol	Electrostatic Energy kcal/mol
X1	+1.1	−37.4	−24.5
X2	−1.4	−38.6	−17.1
X3	−8.2	−48.1	−15.9
X4	−15.3	−40.0	−23.8
X5	+22.8	−25.9	−10.5
X6	−12.0	−51.4	−14.0

**Table 4:** Two-dimensional structures of Benzothiozinone derivatives. * is for the test set.

**C1**	**C2**	C3 *
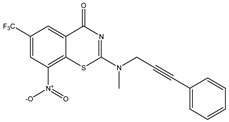	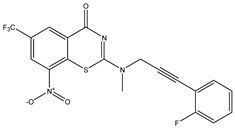	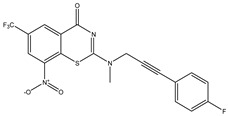
**C4**	C5 *	**C6**
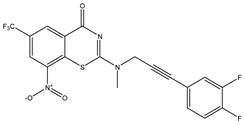	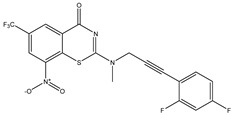	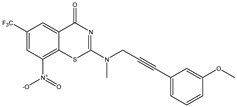
**C7**	**C8**	**C9**
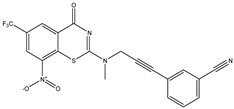	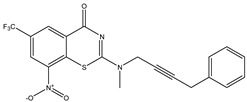	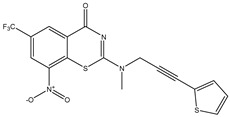
**C10**	C11 *	**C12**
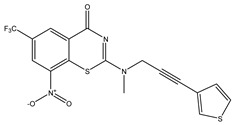	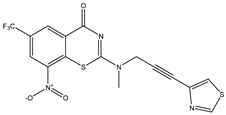	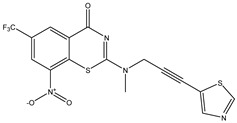
**C13**	**C14**	**C15**
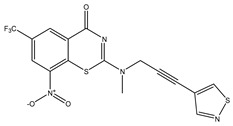	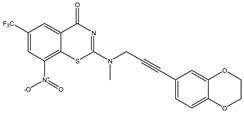	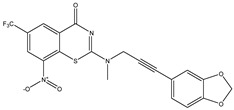
**C16**	**C17**	**C18**
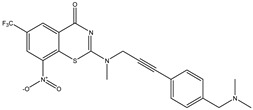	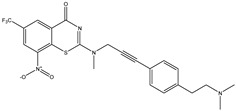	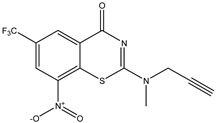
**C19**	C20 *	**C21**
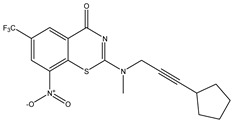	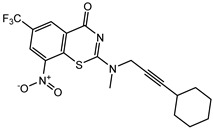	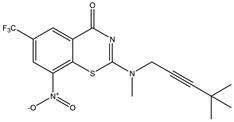
**C22**	**C23**	C24 *
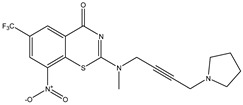	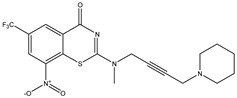	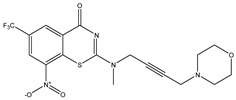
**C25**	**C26**	C27 *
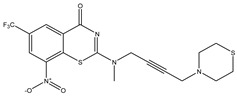	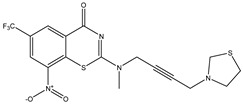	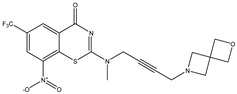
**C28**	**C29**	**C30**
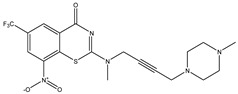	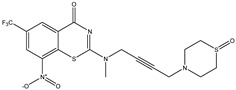	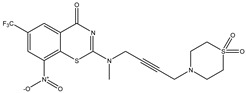
**C31**	**C32**	**C33**
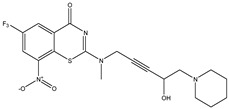	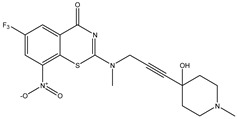	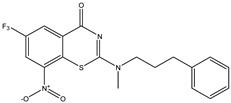
C34 *	C35 *	**C36**
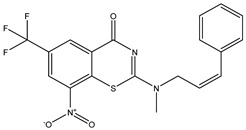	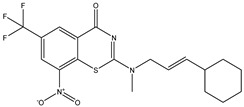	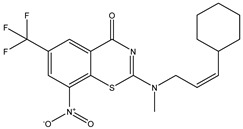
**C37**	**C38**	C39 *
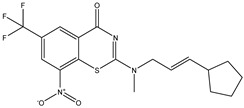	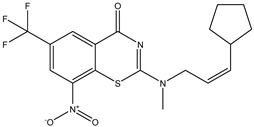	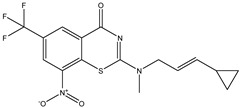
C40 *		
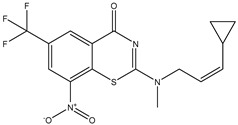		

## Data Availability

All data generated or analyzed during this study are included in this published article.

## Supplementary Materials

The following supporting information can be downloaded at https://www.mdpi.com/article/10.3390/ijms26115129/s1.

## Abbreviations

The following abbreviations are used in this manuscript:

2D-QSAR	Two-dimensional quantitative structure–Activity Relationship
AD	Applicability Domain
ADMET	Absorption, Distribution, Metabolism, Excretion and Toxicity
btz	benzothiozinone
CADD	Computer-Aided Drug Design
HVS	High Virtual Screening
LOO	Leave-One-Out
MD	Molecular Dynamics
MDR	Multidrug-Resistant
MIC	Minimum Inhibitory Concentration
MLR	Multiple Linear Regression
MM-PBSA	Molecular Mechanics Poisson–Boltzmann Surface Area
MMFF	Merck Molecular Force Field
MSE	Mean Squared Error
Mtb	Mycobacterium tuberculosis
PBC	Periodic Boundary Conditions
PDB	Protein DataBank
pMIC	predicted Minimum Inhibitory Concentration
QSAR	Quantitative Structure–Activity Relationship
Rg	Radius of gyration
RMSD	Root Mean Square Deviation
RMSF	Root Mean Square Fluctuation
TB	TuBerculosis
vdW	van der Waals
VS	Virtual Screening
XDR	eXtensively Drug-Resistant
