# Supplementary material for: Virtual Screening of Novel Benzothiozinone Derivatives to Predict Potential Inhibitors of Mycobacterium Tuberculosis Kinases 2D-QSAR, Molecular Docking, MM-PBSA Dynamics Simulations, and ADMET Properties
Source: Int J Mol Sci. 2025 May 27;26(11):5129. doi: 10.3390/ijms26115129 (PMC12155315; doi:10.3390/ijms26115129)
Supplement: Supplementary file 1 [file ijms-26-05129-s001.zip › ijms-3587782-supplementary.pdf]

Supplementary Information

Table S1: MolDock score and H-bond energy of the designed compounds (X21-X48) in the binding pocket of Mtb.

Ligand	MolDock Score kcal/mol	H-Bond kcal/mol
X21	-140.1	-5.3
X22	-139.9	-4.4
X23	-139.5	-4.1
X24	-137.9	-3.7
X25	-136.4	-2.5
X26	-136.2	0.0
X27	-135.6	-3.3
X28	-134.7	-3.0
X29	-132.8	-3.8
X30	-132.2	-2.0
X31	-130.4	-4.5
X32	-129.6	-4.0
X33	-128.9	-6.4
X34	-127.2	-2.5
X35	-126.0	-5.4
X36	-125.5	0.0
X37	-125.3	0.0
X38	-123.1	-2.5
X39	-123.1	-6.8
X40	-122.9	-2.5
X41	-121.6	-5.0
X42	-121.5	-2.5
X43	-120.7	-2.5
X44	-118.4	-4.9
X45	-118.1	-2.5
X46	-116.1	-3.6
X47	-113.9	-2.5
X48	-112.1	-2.5
